# Supplementary material for: Sub-to-super-Poissonian photon statistics in cathodoluminescence of color center ensembles in isolated diamond crystals
Source: Nanophotonics. 2023 May 29;12(12):2231–7. doi: 10.1515/nanoph-2023-0204 (PMC11501345; doi:10.1515/nanoph-2023-0204)
Supplement: Supplementary file 1 — Supplementary Material Details [file j_nanoph-2023-0204_suppl_001.pdf]

Supporting Information: Sub-to-super-Poissonian photon statistics in cathodoluminescence of color center ensembles in isolated diamond crystals

Saskia Fiedler,* Sergii Morozov,* Danylo Komisar, Evgeny A. Ekimov, Liudmila F. Kulikova, Valery A. Davydov, Viatcheslav N. Agafonov, Shailesh Kumar, Christian Wolff, Sergey I. Bozhevolnyi, and N. Asger Mortensen†

Supporting information includes:

- synthesis of color centers in diamond crystals;
- cathodoluminescence spectroscopy;
- optical measurements;
- intensity saturation of a single color center;
- fit of the photon correlation histograms $g_2(\tau)$;
- Fig. S1 electron trajectories in diamond at different accelerating voltages;
- Fig. S2 background emission in diamond crystals;
- Fig. S3 SEM image of as-grown Ge-doped diamond nano-crystals.

Synthesis of color centers in diamond crystals. Germanium- and silicon-doped diamond crystals synthesized by high-pressure methods [26–28]. Ge-doped nano-diamonds with size under 0.7 µm were produced from powder mixture of adamantane $C_{10}H_{16}$ (>99%, Sigma-Aldrich) and tetraphenylgermane $C_{24}H_{20}Ge$ (96%, Sigma-Aldrich) with concentration of Ge 0.08 at % in batch. The reagents were mixed with use of mortar and pestle, both made of poly methyl methacrylate (plexiglas), for about 5 min, pressed into a pellet (55 mg) and placed inside titanium capsule (6 mm in diameter, 4 mm in height, with the 0.2 mm wall thickness). For diamond synthesis, toroid-type high-pressure chamber was used to generate pressure of 9 GPa and temperature between 1600 and 1700 K in the reaction volume. Under pressure, total duration of the heat treatment was about 60 s. After the treatment, the sample was quenched under pressure to room temperature by switching of the electric power. Then, powdered sample was recovered from the capsule and part of it was compressed into Indium plate for SEM investigation (Fig. S3). Sizes of crystals standing alone or being in aggregated form were found to be ranged from 100 to 700 nm.

Diamonds with SiV^- impurity-vacancy color centers were obtained by high pressure - high temperature (HPHT) treatment of the catalyst metal-free hydrocarbon growth system based on homogeneous mixtures of naphthalene, $C_{10}H_8$ (Chemapol), and highly fluorinated graphite, CF1.1 (Aldrich Chemical), and tetrakis(trimethylsilyl)sylane, $C_{12}H_{36}Si_5$ (Stream Chemicals Co.). The synthesis was performed in a high-pressure apparatus of "Toroid" type. Cold-pressed tablets of the initial mixtures (5 mm diameter and 4 mm height) were placed into a graphite container, which simultaneously served as a heater of the high-pressure apparatus. The experimental procedure consisted of loading the high pressure the apparatus up to 8 GPa, heating up to the synthesis temperature ($\sim 1200 - 1400$ ^{o}C) and short isothermal exposure under constant load for 2-10 s. The obtained diamond products were then isolated by quenching to room temperature under pressure. The recovered samples have been characterized by Raman spectroscopy, scanning (SEM) and transmission (TEM) electron microscopies. To purify the sample of traces of non-diamond carbon and excess of Si, it was treated with a mixture of three concentrated acids (HNO_3-$HClO_4$-H_2SO_4) and then with HF respectively. After that, the crystals were washed and dried.

Cathodoluminescence spectroscopy. Cathodoluminescence (CL) spectroscopy is performed in a Tescan Mira3 scanning-electron microscope (SEM) operated at an acceleration voltage between 5 kV and 10 kV, while the electron-beam current is varied from 2.5 pA to 40 pA. Light emitted from the sample is collected by a parabolic mirror and analyzed using a Delmic SPARC CL detector equipped with an Andor Newton charge-coupled device (CCD) camera. All CL spectra are corrected for the system response and the background of the thin SiN membrane. CL maps are collected with activated sub-pixel scanning.

Optical measurements. For the CL second-order correlation measurements, i.e., the $g_2(\tau)$ correlation function as a function of the correlation time τ for the temporal CL intensity $\mathcal{I}(t)$,

$$g_2(\tau) = \frac{\langle \mathcal{I}(t)\mathcal{I}(t+\tau)\rangle}{\langle \mathcal{I}(t)\rangle\langle \mathcal{I}(t+\tau)\rangle} = \frac{\langle \mathcal{I}(t)\mathcal{I}(t+\tau)\rangle}{\langle \mathcal{I}(t)\rangle^2}, \tag{S1}$$

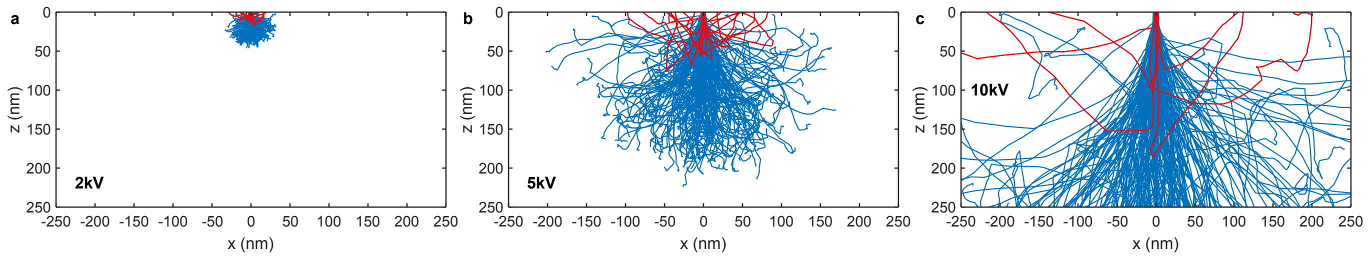

FIG. S1: Electron trajectories in diamond at different accelerating voltages. **a** 2 kV. **b** 5 kV. **c** 10 kV.

a 90:10 beam splitter is inserted before the spectrometer to allow for concurrent spectral and correlation analysis of the sample (see Fig. 1a in the main text). Using a beam splitter, 10% of the CL emission is being transmitted onto the spectrometer, while the remaining 90% is being reflected onto a Hanbury Brown and Twiss (HBT) interferometer. Here, the light beam is split by a 50:50 beam splitter, creating a time delay τ between the incoming photons which are detected via two separated avalanche photodiodes (APDs). The corresponding g_2-data were collected for 60 s, only the measurements at lowest currents (3 pA at 10 kV, and 8 pA at 30 kV) were acquired for 120 s.

It is noteworthy that the CL spectra acquired at the same time as the g_2 measurements exhibit a small spectral shift due to the beam splitter. Therefore, all CL spectra shown in this work have been collected without the beam splitter.

Intensity saturation of a single color center. We fit the intensity saturation in Fig. 3d of the main text using the equation

$$C = C_\infty \frac{I}{I + I_{\text{sat}}}, \tag{S2}$$

where C is the emission count rate, I is the electron beam current, and C_∞ and I_{sat} are the emission rate and electron beam current at saturation, respectively.

Fit of the photon-correlation histograms $g_2(\tau)$. We fit the anti-bunching and bunching histograms using a general equation describing a bi-exponential function describing the slow and fast components of the central peak at zero correlation times and an additional time constant for the recombination through the shelving state.

The general form of $g_2(\tau)$ for arbitrary number of emitters N assumes a bi-exponential dynamics (fast and slow time constants τ_1 and τ_2, respectively) of the central peak at zero correlation times as well as an additional time constant τ_{sh} for the recombination through the shelving state. The amplitudes of the recombination pathways are described by two coefficients a and c, where a defines the recombination through the shelving state, and c accounts for the recombination through the slow process with lifetime τ_2:

$$g_2(\tau, N) = 1 - \left(a + \frac{1}{N}\right) e^{-\frac{|\tau|}{\tau_e}} + \frac{I_0}{I}\left(1 - \frac{1}{N}\right) e^{-\frac{|\tau|}{\tau_e}} + a e^{-\frac{|\tau|}{\tau_{\text{sh}}}}. \tag{S3}$$

Here, τ is the delay time, I_0 is the minimal current required for the high energy inter-band excitation.

In case of a single color center ($N = 1$) with a shelving state, the equation Eq. S3 converges to one which describes emission dynamics of three-level system at increasing laser power [33]

$$g_2(\tau, N = 1) = 1 - (a + 1)\, e^{-\frac{|\tau|}{\tau_e}} + a e^{-\frac{|\tau|}{\tau_{\text{sh}}}}. \tag{S4}$$

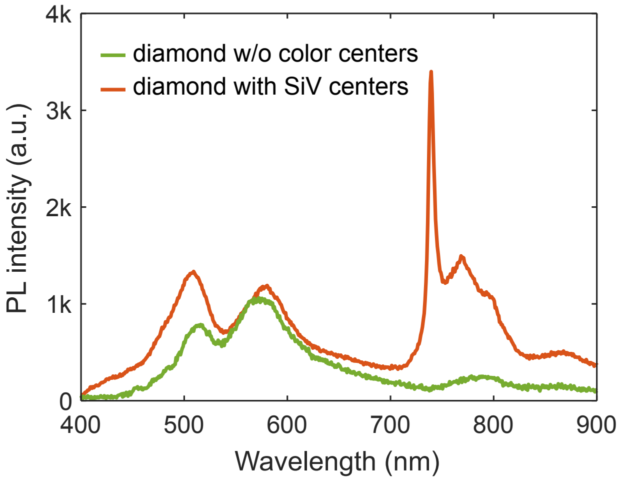

FIG. S2: Background emission in diamond crystals. Spectra acquired from a crystal with (orange) and without (green) color centers. The background originates from impurities in diamond and Mie-excited resonances.

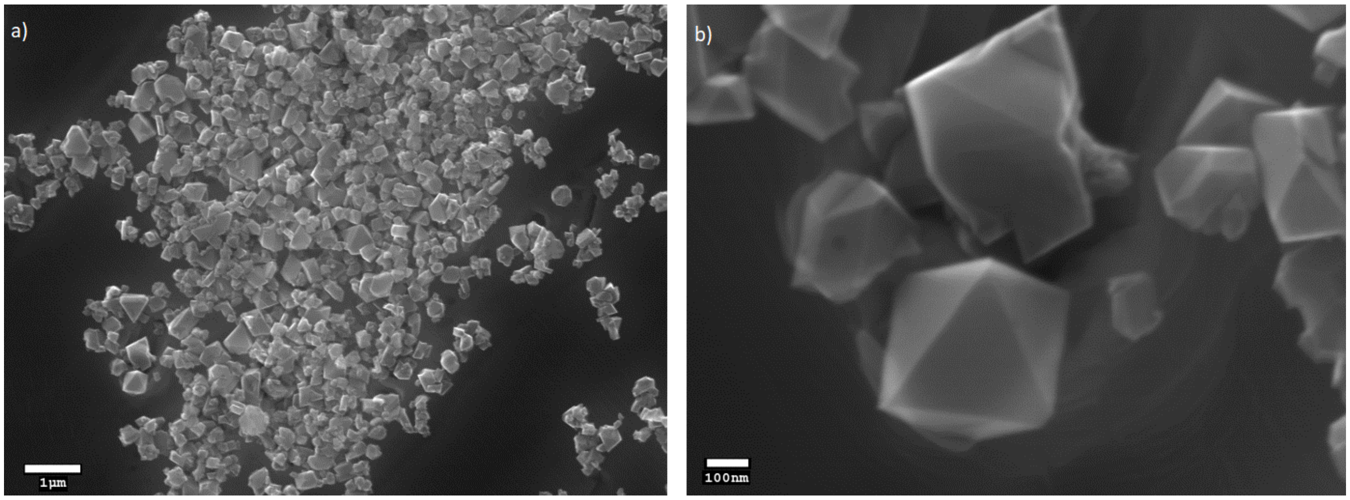

FIG. S3: SEM image of as-grown Ge-doped diamond nano-crystals. **a** Widefield SEM image of aggregated nano-crystals. **b** High-resolution SEM image with single nano-crystals.

---

* S. M. and S. F. contributed equally to this work.
† Corresponding author

[1] S. Meuret, L. H. G. Tizei, T. Cazimajou, R. Bourrellier, H. C. Chang, F. Treussart, and M. Kociak, "Photon bunching in cathodoluminescence," Phys. Rev. Lett., vol. 114, no. 19, p. 197401, 2015.

[2] P. Dombi, Z. Pápa, J. Vogelsang, S. V. Yalunin, M. Sivis, G. Herink, S. Schäfer, P. Groß, C. Ropers, and C. Lienau, "Strong-field nano-optics," Rev. Mod. Phys., vol. 92, no. 2, p. 025003, 2020.

[3] I. Aharonovich, D. Englund, and M. Toth, "Solid-state single-photon emitters," Nat. Photon., vol. 10, no. 10, pp. 631–641, 2016.

[4] F. J. García de Abajo, "Optical excitations in electron microscopy," Rev. Mod. Phys., vol. 82, no. 1, pp. 209–275, 2010.

[5] A. Polman, M. Kociak, and F. J. García de Abajo, "Electron-beam spectroscopy for nanophotonics," Nat. Mater., vol. 18, no. 11, pp. 1158–1171, 2019.

[6] J. Nelayah, M. Kociak, O. Stéphan, F. J. García de Abajo, M. Tencé, L. Henrard, D. Taverna, I. Pastoriza-Santos, L. M. Liz-Marzán, and C. Colliex, "Mapping surface plasmons on a single metallic nanoparticle," Nat. Phys., vol. 3, no. 5, pp. 348–353, 2007.

[7] H. Duan, A. I. Fernández-Domínguez, M. Bosman, S. A. Maier, and J. K. W. Yang, "Nanoplasmonics: classical down to the nanometer scale," Nano Lett., vol. 12, no. 3, pp. 1683–1689, 2012.

[8] J. A. Scholl, A. L. Koh, and J. A. Dionne, "Quantum plasmon resonances of individual metallic nanoparticles," Nature,

vol. 483, no. 7390, p. 421, 2012.

[9] S. Raza, N. Stenger, A. Pors, T. Holmgaard, S. Kadkhodazadeh, J. B. Wagner, K. Pedersen, M. Wubs, S. I. Bozhevolnyi, and N. A. Mortensen, “Extremely confined gap surface-plasmon modes excited by electrons,” Nat. Commun., vol. 5, p. 4125, 2014.

[10] A. Losquin, L. F. Zagonel, V. Myroshnychenko, B. Rodríguez-González, M. Tencé, L. Scarabelli, J. Förstner, L. M. Liz-Marzán, F. J. García de Abajo, O. Stéphan, and M. Kociak, “Unveiling nanometer scale extinction and scattering phenomena through combined electron energy loss spectroscopy and cathodoluminescence measurements,” Nano Lett., vol. 15, no. 2, pp. 1229–1237, 2015.

[11] A. Campos, N. Troc, E. Cottancin, M. Pellarin, H.-C. Weissker, J. Lermé, M. Kociak, and M. Hillenkamp, “Plasmonic quantum size effects in silver nanoparticles are dominated by interfaces and local environments,” Nat. Phys., vol. 15, no. 3, pp. 275–280, 2019.

[12] T. Sannomiya, A. Konečná, T. Matsukata, Z. Thollar, T. Okamoto, F. J. García de Abajo, and N. Yamamoto, “Cathodoluminescence phase extraction of the coupling between nanoparticles and surface plasmon polaritons,” Nano Lett., vol. 20, no. 1, pp. 592–598, 2020.

[13] S. Fiedler, P. E. Stamatopoulou, A. Assadillayev, C. Wolff, H. Sugimoto, M. Fujii, N. A. Mortensen, S. Raza, and C. Tserkezis, “Disentangling cathodoluminescence spectra in nanophotonics: Particle eigenmodes vs transition radiation,” Nano Lett., vol. 22, no. 6, pp. 2320–2327, 2022.

[14] N. Varkentina, Y. Auad, S. Y. Woo, A. Zobelli, L. Bocher, J.-D. Blazit, X. Li, M. Tencé, K. Watanabe, T. Taniguchi, O. Stéphan, M. Kociak, and L. H. G. Tizei, “Cathodoluminescence excitation spectroscopy: Nanoscale imaging of excitation pathways,” Sci. Adv., vol. 8, no. 40, p. eabq4947, 2022.

[15] S. Fiedler, S. Raza, R. Ai, J. Wang, K. Busch, N. Stenger, N. A. Mortensen, and C. Wolff, “Importance of substrates for the visibility of “dark” plasmonic modes,” Opt. Express, vol. 28, no. 9, pp. 13 938–13 948, 2020.

[16] S. Mignuzzi, M. Mota, T. Coenen, Y. Li, A. P. Mihai, P. K. Petrov, R. F. M. Oulton, S. A. Maier, and R. Sapienza, “Energy–momentum cathodoluminescence spectroscopy of dielectric nanostructures,” ACS Photonics, vol. 5, no. 4, pp. 1381–1387, 2018.

[17] L. H. G. Tizei and M. Kociak, “Spatially resolved quantum nano-optics of single photons using an electron microscope,” Phys. Rev. Lett., vol. 110, no. 15, p. 153604, 2013.

[18] R. Bourrellier, S. Meuret, A. Tararan, O. Stéphan, M. Kociak, L. H. G. Tizei, and A. Zobelli, “Bright UV single photon emission at point defects in h-BN,” Nano Lett., vol. 16, no. 7, p. 4317–4321, 2016.

[19] M. A. Feldman, E. F. Dumitrescu, D. Bridges, M. F. Chisholm, R. B. Davidson, P. G. Evans, J. A. Hachtel, A. Hu, R. C. Pooser, R. F. Haglund, and B. J. Lawrie, “Colossal photon bunching in quasiparticle-mediated nanodiamond cathodoluminescence,” Phys. Rev. B, vol. 97, no. 8, p. 081404(R), 2018.

[20] S. Meuret, T. Coenen, H. Zeijlemaker, M. Latzel, S. Christiansen, S. Conesa-Boj, and A. Polman, “Photon bunching reveals single-electron cathodoluminescence excitation efficiency in InGaN quantum wells,” Phys. Rev. B, vol. 96, no. 3, p. 035308, 2017.

[21] S. Fiedler, S. Morozov, L. Iliushyn, S. Boroviks, M. Thomaschewski, J. Wang, T. J. Booth, N. Stenger, C. Wolff, and N. A. Mortensen, “Photon superbunching in cathodoluminescence of excitons in WS_2 monolayer,” 2D Materials, vol. 10, no. 2, p. 021002, 2023.

[22] R. F. Egerton, Electron energy-loss spectroscopy in the electron microscope. New York: Springer, 2011.

[23] S. Meuret, “Applications of photon bunching in cathodoluminescence,” Adv. Imaging Electron Phys., vol. 215, pp. 47–87, 2020.

[24] V. V. Temnov and U. Woggon, “Photon statistics in the cooperative spontaneous emission,” Opt. Express, vol. 17, no. 7, pp. 5774–5782, 2009.

[25] T. Yuge, N. Yamamoto, T. Sannomiya, and K. Akiba, “Superbunching in cathodoluminescence: A master equation approach,” Phys. Rev. B, vol. 107, p. 165303, 2023.

[26] E. A. Ekimov, M. V. Kondrin, V. S. Krivobok, A. A. Khomich, I. I. Vlasov, R. A. Khmelnitskiy, T. Iwasaki, and M. Hatano, “Effect of Si, Ge and Sn dopant elements on structure and photoluminescence of nano- and microdiamonds synthesized from organic compounds,” Diam. Relat. Mater., vol. 93, pp. 75–83, 2019.

[27] K. M. Kondrina, O. S. Kudryavtsev, I. I. Vlasov, R. A. Khmelnitskiy, and E. A. Ekimov, “High-pressure synthesis of microdiamonds from polyethylene terephthalate,” Diam. Relat. Mater., vol. 83, no. 190, pp. 190–195, 2018.

[28] V. A. Davydov, A. V. Rakhmanina, S. G. Lyapin, I. D. Ilichev, K. N. Boldyrev, A. A. Shiryaev, and V. N. Agafonov, “Production of nano- and microdiamonds with Si-V and N-V luminescent centers at high pressures in systems based on mixtures of hydrocarbon and fluorocarbon compounds,” JETP Lett., vol. 99, no. 10, pp. 585–589, 2014.

[29] D. Drouin, A. R. Couture, D. Joly, X. Tastet, V. Aimez, and R. Gauvin, “CASINO V2.42–a fast and easy-to-use modeling tool for scanning electron microscopy and microanalysis users,” Scanning, vol. 29, no. 3, pp. 92–101, 2007.

[30] C. Bradac, W. Gao, J. Forneris, M. E. Trusheim, and I. Aharonovich, “Quantum nanophotonics with group IV defects in diamond,” Nat. Commun., vol. 10, p. 5625, 2019.

[31] H. Takashima, A. Fukuda, K. Shimazaki, Y. Iwabata, H. Kawaguchi, A. W. Schell, T. Tashima, H. Abe, S. Onoda, T. Ohshima, and S. Takeuchi, “Creation of silicon vacancy color centers with a narrow emission line in nanodiamonds by ion implantation,” Opt. Mater. Express, vol. 11, no. 7, p. 1978, 2021.

[32] S. Lagomarsino, A. M. Flatae, H. Kambalathmana, F. Sledz, L. Hunold, N. Soltani, P. Reuschel, S. Sciortino, N. Gelli, M. Massi, C. Czelusniak, L. Giuntini, and M. Agio, “Creation of silicon-vacancy color centers in diamond by ion implantation,” Front. Phys., vol. 8, p. 601362, 2021.

[33] M. Nahra, D. Alshamaa, R. Deturche, V. Davydov, L. Kulikova, V. Agafonov, and C. Couteau, “Single germanium vacancy

centers in nanodiamonds with bulk-like spectral stability," AVS Quantum Science, vol. 3, no. 1, p. 012001, 2021.

[34] S. Prawer and I. Aharonovich, Eds., Quantum information processing with Diamond, ser. Woodhead Publishing Series in Electronic and Optical Materials. Cambridge, England: Woodhead Publishing, May 2014.

[35] M. Solà-Garcia, K. W. Mauser, M. Liebtrau, T. Coenen, S. Christiansen, S. Meuret, and A. Polman, "Photon statistics of incoherent cathodoluminescence with continuous and pulsed electron beams," ACS Photonics, vol. 8, no. 3, pp. 916–925, 2021.

AUTHOR INFORMATION

S. Fiedler orcid.org/0000-0002-7753-0814
S. Morozov orcid.org/0000-0002-5415-326X
D. Komisar orcid.org/0000-0001-8856-7586
L. F. Kulikova orcid.org/0000-0002-9070-0590
V. A. Davydov orcid.org/0000-0002-8702-0340
V. N. Agafonov orcid.org/0000-0001-5770-1252
E. A. Ekimov orcid.org/0000-0001-7644-0078
S. Kumar orcid.org/0000-0001-5795-0910
C. Wolff orcid.org/0000-0002-5759-6779
S. I. Bozhevolnyi orcid.org/0000-0002-0393-4859
N. A. Mortensen orcid.org/0000-0001-7936-6264
